# Assessment of Proficiency of N95 Mask Donning Among the General Public in Singapore

**DOI:** 10.1001/jamanetworkopen.2020.9670

**Published:** 2020-05-20

**Authors:** Wesley Yeung, Kennedy Ng, J. M. Nigel Fong, Judy Sng, Bee Choo Tai, Sin Eng Chia

**Affiliations:** 1 University Medicine Cluster, National University Hospital, Singapore; 2 Laboratory for Computational Physiology, Harvard-MIT Health Sciences and Technology, Massachusetts Institute of Technology, Cambridge; 3 Division of Medical Oncology, National Cancer Centre Singapore, Singapore; 4 Division of Medicine, Singapore General Hospital, Singapore; 5 Saw Swee Hock School of Public Health, National University of Singapore, Singapore

## Abstract

This cross-sectional study examines factors associated with proper use of N95 masks among residents of Singapore.

## Introduction

With the advent of the coronavirus disease 2019 (COVID-19) pandemic, mask-wearing among the general public has become commonplace, leading to a worldwide shortage.^1^ However, there is little data on mask-wearing proficiency in the general public. A single study performed in the US after Hurricane Katrina, when individuals donned masks for mold remediation, found that only 24% of participants demonstrated proper technique.^2^ Incorrectly worn masks may not confer effective protection against COVID-19.

We conducted this cross-sectional study to evaluate the proficiency of members of the Singapore public in wearing N95 masks, which the local government distributed to households in 2014 as part of an emergency preparedness program^3^ targeted at episodes of haze. The duck-bill foldable N95 mask (3M VFlex 9105) was selected for ease of mailing and was distributed along with pictorial instructions.

## Methods

The institutional review board of the National University of Singapore granted approval for our study. Verbal informed consent was obtained from all participants, and a participant information sheet was provided. This study is reported following Strengthening the Reporting of Observational Studies in Epidemiology (STROBE) reporting guideline.

We conducted a cross-sectional study in the Jurong district in Singapore from February 9 to 15, 2015. Participants were recruited by simple random sampling without replacement. Inclusion criteria were Singapore citizens or permanent residents aged 21 years or older who lived in Singapore in June through July 2013 (during a severe episode of transboundary haze) and who were physically able to independently don the N95 mask. N95 masks, with accompanying multilingual pictorial instructions, were given to respondents. These masks and instruction sheets were of the same model as those mailed out to Singapore residents in 2014. The participant was then asked to put on the mask, with no prompting to refer to the instruction sheet. Interviewers administered a visual mask fit (VMF) test^2^ and a user seal check following the manufacturer’s instructions.^4^ Our primary outcome was passing the VMF test.

Pearson χ^2^ tests and Welch *t* test were used for bivariate analyses, and a log-binomial regression model was used for multivariable analyses. Data were analyzed using R statistical software (R Project for Statistical Computing). *P* values were 2-sided, and statistical significance was set at .05. Analysis was conducted from February to March 2015.

## Results

The survey was administered to 2499 households. Of these, 268 households were excluded because they did not meet the inclusion criteria. Among the remaining 2231 households, 714 (32.0%) completed the survey, 541 (24.2%) declined to be surveyed, and 976 (43.7%) did not respond. There were slightly more women participants (382 women [53.5%]). Most participants were aged 41 to 65 years (356 participants [49.9%]) and were of Chinese ethnicity (514 participants [72.0%]) (Table 1). Only 90 participants (12.6%; 95% CI, 10.3%-15.3%) passed the VMF test. The most common mask-fit criteria performed incorrectly were strap placement (521 participants [73.0%; 95% CI, 69.6%-76.2%]), leaving a visible gap between the mask and skin (442 participants [61.9%; 95% CI, 58.2%-65.5%]), and tightening the nose-clip (431 participants [60.4%; 95% CI, 56.7%-64.0%]). Younger age (adjusted prevalence ratio per 1-year increase in age, 0.95; 95% CI, 0.94-0.96; *P* < .001) and previous mask-fit training (adjusted prevalence ratio, 2.25; 95% CI, 1.54-3.30; *P* < .001) were independently associated with higher pass rates (Table 2). The use of the instruction leaflet provided, ownership of N95 masks, and previous mask use were not significantly associated with passing the VMF test.

**Table 1.** Demographic Characteristics of Study Participants

Characteristic	No. (%) (N = 714)
Sex	
Men	332 (46.5)
Women	382 (53.5)
Age, y	
21-40	242 (33.9)
41-65	356 (49.9)
>65	116 (16.2)
Ethnicity	
Chinese	514 (72.0)
Indian	106 (14.8)
Malay	86 (12.0)
Others	8 (1.1)
Type of residence	
Rental	33 (4.6)
Self-owned, rooms	
1-2	12 (1.7)
3	192 (26.9)
4	248 (34.7)
≥5	229 (32.1)
Education	
None	31 (4.3)
Primary	119 (16.7)
Secondary	213 (29.8)
Tertiary	183 (25.6)
University	168 (23.5)
Monthly household income, SGD	
None	139 (19.5)
<3000 (US $2117.30)	100 (14.0)
3000-5000 (US $2117.30-$3528.83)	166 (23.2)
>5000-8000( US $3528.83-$5646.12)	120 (16.8)
>8000-12000 (US $5646.13-$8469.19)	113 (15.8)
>12000 (US $8469.19)	58 (8.1)
Unknown	18 (2.5)
Employment status^a^	
Employed	425 (59.5)
Unemployed	288 (40.3)

Abbreviation: SGD, Singapore dollars.

^a^ Missing data for 1 participant.

**Table 2.** Bivariate and Multivariable Analysis of Factors Associated With Visual Mask Fit Test Pass

Characteristic	No. (%)		Unadjusted prevalence ratio (95% CI)	*P* value	Adjusted prevalence ratio (95% CI)	*P* value
	Passed (n = 90)	Failed (n = 624)				
Age, mean (SD), y	41.4 (13.8)	50.2 (16.3)	NA	<.001	0.95 (0.94-0.96)^a^	<.001
Read manufacturer’s instructions	23 (25.6)	142 (22.8)	1.14 (0.74-1.77)	.56	0.75 (0.46-1.25)	.27
Owned mask previously	80 (88.9)	478 (76.6)	2.24 (1.19-4.21)	.008	1.46 (0.82-2.60)	.20
Used mask previously	46 (51.1)	264 (42.3)	1.36 (0.93-2.00)	.12	0.80 (0.49-1.33)	.39
Received prior mask fit training	42 (46.7)	118 (18.9)	3.03 (2.08-4.41)	<.001	2.25 (1.54-3.30)	<.001

Abbreviation: NA, not applicable.

^a^ Per 1-year increase.

## Discussion

Our study found a low N95 VMF pass rate of 12.6%. The observation that reading pictorial instructions was not associated with increased VMF pass rates may suggest an inherent complexity to N95 mask wearing. Limitations of this study include nonresponse and recall bias.

These findings support ongoing recommendations against the use of N95 masks by the general public during the COVID-19 pandemic.^5^ N95 mask use by the general public may not translate into effective protection but instead provide false reassurance. Beyond N95 masks, proficiency among the general public in donning surgical masks needs to be assessed. Policy measures that encourage mask use in the general public must be coupled with effective training materials beyond instruction leaflets, which our study and a 2013 study by Harber et al^6^ found to be inadequate. Other public health measures, such as social distancing, handwashing, and self-isolation when ill, are also critical.
